# Universal principles justify the existence of concept cells

**DOI:** 10.1038/s41598-020-64466-7

**Published:** 2020-05-12

**Authors:** Carlos Calvo Tapia, Ivan Tyukin, Valeri A. Makarov

**Affiliations:** 1 Instituto de Matemática Interdisciplinar, Faculty of Mathematics, Universidad Complutense de Madrid, Plaza de Ciencias 3, Madrid, 28040 Spain; 2 University of Leicester, Department of Mathematics, University Road, LE1 7RH United Kingdom; 3 Lobachevsky University of Nizhny Novgorod, Gagarin Ave. 23, Nizhny, Novgorod 603950 Russia

**Keywords:** Biological physics, Nonlinear phenomena

## Abstract

The widespread consensus argues that the emergence of abstract concepts in the human brain, such as a “table”, requires complex, perfectly orchestrated interaction of myriads of neurons. However, this is not what converging experimental evidence suggests. Single neurons, the so-called concept cells (CCs), may be responsible for complex tasks performed by humans. This finding, with deep implications for neuroscience and theory of neural networks, has no solid theoretical grounds so far. Our recent advances in stochastic separability of highdimensional data have provided the basis to validate the existence of CCs. Here, starting from a few first principles, we layout biophysical foundations showing that CCs are not only possible but highly likely in brain structures such as the hippocampus. Three fundamental conditions, fulfilled by the human brain, ensure high cognitive functionality of single cells: a hierarchical feedforward organization of large laminar neuronal strata, a suprathreshold number of synaptic entries to principal neurons in the strata, and a magnitude of synaptic plasticity adequate for each neuronal stratum. We illustrate the approach on a simple example of acquiring “musical memory” and show how the concept of musical notes can emerge.

## Introduction

Brains are undoubtedly high-dimensional^1,2^. Even the simplest animal, the rotifer $0.5$ mm long, has 200 neurons acting in parallel as coupled dynamical systems, while in the human brain, this figure rises to billions. Such a huge range of the number of neurons in different species has been related to the great variety of their cognitive abilities^3,4^.

Here, however, we assess the implication of another brain dimension, the number of synaptic inputs, $n$, a single neuron receives. Recent empirical evidence shows that a variation in the dendrite length and hence in the number of synapses $n$ can explain up to $\mathrm{25 \% }$ of the variance in IQ scores between individuals^5^. However, no rigorous biophysical theory explaining how $n$ affects high-level cognitive abilities has been put forward yet.

The importance of such a theory and the underlying universal principles is difficult to overestimate. For example, the design of modern artificial neural networks (ANNs) copies the converging architecture of biological sensory systems^6^. As a result, they already outperform humans in pattern recognition benchmarks yet remaining far behind in cognition^7,8^. Thus, the next qualitative leap in the development of ANNs requires novel biophysical insights on the functional architecture and dynamical principles of higher brain stations.

A step towards may reside in recent mathematical studies of the so-called *“grandmother” cells*^1,9^. Converging experimental evidence suggests that some pyramidal neurons in the medial temporal lobe (MTL) can exhibit remarkable selectivity and invariance to complex stimuli. In particular, it has been shown that the so-called *concept cells* (CCs) can fire when a subject sees one of seven different pictures of Jennifer Aniston but not the other 80 pictures of other persons and places^10^. CCs can also fire to the spoken or written name of the same person^11^. Thus, a single CC responds to an abstract concept but not to the sensory features of the stimuli. This empirical observation casts doubts on the widespread belief that complex cognitive phenomena require the perfectly orchestrated collaboration of many neurons. Moreover, CCs are relatively easily recorded in the hippocampus^12^. Thus, they must be abundant, at least in the MTL, contrary to the common opinion that their existence is highly unlikely^13^. Nevertheless, the experimental approach cannot fully isolate the single-cell contribution to the network dynamics, and a theoretical study of the biological mechanisms underlying CCs is required.

Presumably, CCs play a role in episodic memory^11^. Memory formation and retrieval have been in the center of attention for several decades, starting from the seminal Hopfield’s work^14^. Recently, the linear scaling of the memory capacity with a low factor of $0.14$ has been overcome^15^. Yet, as has been found, memory retrieval is inherently unstable due to complex network dynamics^16^. Thus, a “single”-neuron approach to memory functions, likely implemented by CCs, can also be useful from both theoretical and experimental viewpoints.

Early, a purely statistical approach for mimicking the sparse coding in the brain has been proposed^17,18^. Using a problem-tailored sparse distribution of categories and an unsupervised expectation-maximization of a log-likelihood functional, Waydo and Koch showed that a network of nonlinear units can map input images to categories^18^. Such a mapping displays sparse invariant selectivity similar to the data observed in the MTL. However, the fundamental questions how and why it happens in the brain have not been addressed yet.

In this work, we report the first theoretical justification of the existence of CCs. Our approach involves a few first biophysical principles and uses the neuronal dimension $n$ as the major factor. We suggest that the evolution of neurons led to an increase of their input dimension $n$, which triggered a qualitative leap in their function and emergence of CCs, and episodic memory.

## Methods

### Model architecture

**Figure 1:**
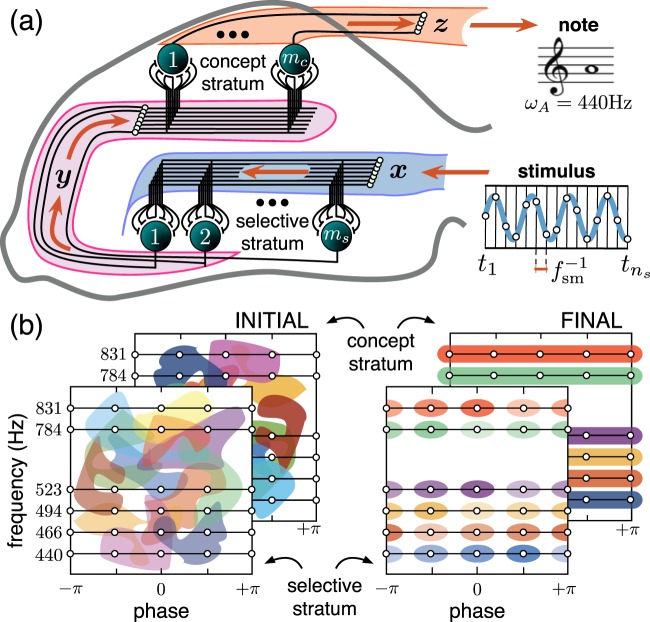
Hypothesis of concept cells. **(a)** Model mimicking the information flow in the hippocampus. A stimulus, a sound wave in the example, activates the concept of a musical note. **(b)** Rearrangement of the neuronal “receptive fields” leads to the formation of note-specific concept cells (different colors correspond to the receptive fields of different neurons).

Figure 1(a) illustrates the model mimicking primary signaling pathways in the hippocampus. It takes into account the stratified structure of the hippocampus that facilitates ramification of axons, leaving multiple buttons in passage and hence conveying the same high-dimensional input to multiple pyramidal cells. The latter has been supported by electrophysiological observations showing that Schaffer collaterals create modules of coherent activity with a large spatial extension in the CA3 region^19,20^. Thus, the hippocampal formation possesses rather exclusive anatomical and functional properties required for the emergence of concept cells, as we discuss below.

For simplicity, we consider one “selective” and one “concept” neuronal strata only and neglect the connections between neurons within each layer. Moreover, as we will see below, the network is randomly initialized, and learning is localized within individual neurons. It is unsupervised, and hence no global fitness function usually used in the ANN approach is required. Thus, the model discards all *a priori* assumptions on the local network structure and dynamics.

The first (selective) stratum contains ${m}_{s}$ neurons receiving in a sequence $L$ ${n}_{s}$-dimensional (${n}_{s}$ D) stimuli ${{\bf{x}}}_{i}\in {[-\mathrm{1,1]}}^{{n}_{s}}$ ($i=\mathrm{1,2,}\ldots ,L$), e.g., sound waves (Fig. 1(a)). In general, ${m}_{s}\gg L$ (e.g., in the CA1 region of the hippocampus, there are $1.4\times {10}^{7}$ pyramidal cells). The output from the first stratum ${{\bf{y}}}_{i}$, as a response to stimulus ${{\bf{x}}}_{i}$, goes to the second stratum consisting of ${m}_{c}$ neurons. We assume that each neuron in the concept stratum receives input from all neurons of the selective stratum. Therefore, the input dimension of the neurons in the concept stratum is equal to the number of neurons in the selective stratum ${n}_{c}={m}_{s}$. In the concept stratum, $K$ consecutive signals $\{{{\bf{y}}}_{i}\}$ can overlap in time due to short-term memory, implemented through, e.g., synaptic integration, and we get output ${\bf{z}}$, which codifies concepts (musical notes in Fig. 1(a)).

We note that neurons in the concept stratum associate several items and then respond to groups of stimuli, which form concepts (in our case, concepts of musical notes). Stimuli within a group can be uncorrelated and even represent different sensory modalities, which gives rise to complex concepts as experimentally observed^11^.

### Stimuli and concepts

Although the nature of stimuli ${{\bf{x}}}_{i}$ can be arbitrary, we illustrate the model on a simple example of acquiring “musical memory”. To follow the music, the system must be able to recognize the tones of sound or notes unambiguously. To preserve generality, we do not apply any algorithmic pre-processing of sound signals, largely extended in ANNs. A piece of a sound wave sampled at ${f}_{{\rm{s}}{\rm{m}}}={2}^{13}$ Hz can be represented as a “raw” ${n}_{s}$ D stimulus (Fig. 1(a)):

1 $${{\bf{x}}}_{i}=({A}_{i}\,\cos \,(2\pi {f}_{i}t/{f}_{{\rm{s}}{\rm{m}}}+{\phi }_{i}{))}_{t=1}^{{n}_{s}},$$

where ${A}_{i}$, ${f}_{i}$, and ${\phi }_{i}$ are the amplitude, frequency, and phase, respectively. Let’s assume that ${A}_{i}$ is fixed, i.e., the amplitude is normalized by a sensory organ. Then, $\Omega =\{(f,\phi )\}$ defines the set of primary stimuli. In this set, for example, musical note A corresponds to frequency $440$ Hz, i.e., to the subset ${\omega }_{A}=\{(440,\,\phi )\,:\,{\rm{\forall }}\phi \}\subset \Omega$.

At the beginning, all neurons in both strata are initialized randomly. Therefore, their “receptive fields” (areas in the sensory domain $\Omega$ invoking a response of a neuron) form a disordered mixture of random regions (see the cartoon in Fig. 1(b), left). Thus, the output of the concept stratum is random, and the system cannot follow the music. The purpose of learning is to organize the receptive fields in such a way that the concept cells become note-specific, i.e., they should fire in response to a given tone regardless of its phase (Fig. 1(b), right). In this case, each concept cell will not be a stimulus-specific but represent a set of associated stimuli or a concept, e.g., note A.

To enable such learning, we need at least a two-stratum system. Neurons in the selective stratum learn to respond selectively to all sound waves, while neurons in the concept stratum associate stimuli with different phases but with the same frequency. Such an association cannot be done within the first stratum, since raw signals can be anti-correlated, e.g., ${\phi }_{1}=0$ and ${\phi }_{2}=\pi$ in Eq. (1), and then cancel each other on a neuron ${{\bf{x}}}_{1}+{{\bf{x}}}_{2}=0$.

### Neuronal dynamics

All neurons in both strata are described by the same model^1,9^, which captures the threshold nature of the neuronal activation but disregards the dynamics of spike generation. The response of the *j*-th neuron ${y}_{j}(t)$ in the selective stratum to the external input ${{\bf{s}}}_{{\rm{e}}{\rm{x}}{\rm{t}}}(t)$ is given by:

2a $${{\bf{s}}}_{{\rm{e}}{\rm{x}}{\rm{t}}}=\mathop{\sum }\limits_{i=1}^{L}\sum _{k}\sqrt{\frac{3}{{n}_{s}}}{{\bf{x}}}_{i}{\sigma }_{ik}(t),$$

2b $${y}_{j}=H({v}_{j}-{\theta }_{j}),\,{v}_{j}=\langle {{\bf{w}}}_{j},{{\bf{s}}}_{{\rm{ext}}}\rangle ,$$

2c $${\dot{{\bf{w}}}}_{j}=\alpha {y}_{j}({\beta }^{2}{{\bf{s}}}_{{\rm{ext}}}-{v}_{j}{{\bf{w}}}_{j}),$$

where ${\sigma }_{ik}(t)$ are disjoint rectangular time windows defining the *k*-th appearance of the *i*-th stimulus, $H(u)=\,{\rm{\max }}\,\mathrm{\{0,}\,u\}$ is the transfer function, ${v}_{j}(t)$ is the membrane potential, ${\theta }_{j}\ge 0$ is the “firing” threshold, ${{\bf{w}}}_{j}(t)\in {{\mathbb{R}}}^{{n}_{s}}$ is the vector of the synaptic weights, $\langle \cdot \,,\,\cdot \rangle$ is the standard inner product, $\alpha  > 0$ defines the relaxation time, and $\beta  > 0$ is an order parameter that will be defined later.

Equation (2c) simulates the Hebbian type of synaptic plasticity. The term proportional to ${y}_{j}{{\bf{s}}}_{{\rm{ext}}}$ forces plastic changes when a stimulus evokes a non-zero neuronal response only, similar to the classical Oja rule^21^. The second term ensures boundness of ${{\bf{w}}}_{j}$ to conform with physical plausibility.

## Results

We now assess the implication of the neuronal input dimensions in the selective and concept strata (${n}_{s}$ and ${n}_{c}$) on the emergence of concept cells.

### Emergence of extreme selectivity

Since we assume no *a priory* information, at $t=0$, the synaptic weights of all neurons, ${{\bf{w}}}_{j}\mathrm{(0)}$, are randomly initialized in the hypercube ${U}^{{n}_{s}}([\,-\,\mathrm{1,1])}$. The threshold values ${\theta }_{j}$ can also be chosen arbitrary. Then, neuron $j$ “fires” in response to stimulus ${{\bf{x}}}_{i}$ if its membrane potential exceeds the threshold, ${v}_{j} > {\theta }_{j}$. In this case, we say that the neuron *detects* the stimulus. Let ${d}_{j}\in \mathrm{\{0,}\,\mathrm{1,}\ldots ,L\}$ be the number of stimuli the *j*-th neuron can detect. Then, if ${d}_{j}=0$, the neuron is *inactive* for the given stimulus set, it is *selective* if ${d}_{j}=1$, and non-selective otherwise.

To quantify the performance of the selective stratum, we introduce the ratios of selective neurons ${R}_{{\rm{slctv}}}$, i.e., the number of selective neurons over ${m}_{s}$; inactive neurons ${R}_{{\rm{inact}}}$, i.e., the number of inactive neurons over ${m}_{s}$; and *“lost”* stimuli ${R}_{{\rm{lost}}}$, i.e., the number of stimuli that excite no neurons over $L$.

To estimate the expected values of these indexes, we note that a random stimulus ${{\bf{x}}}_{i}$, taken from ${U}^{{n}_{s}}([-\mathrm{1,1])}$, elicits random membrane potential in each neuron, which will be normally distributed as $v\sim {\mathscr{N}}\mathrm{(0,}\frac{1}{\sqrt{3}})$, up to an error term of order $O\mathrm{(1}/\sqrt{{n}_{s}})$. For ${n}_{s}$ large enough (${n}_{s}\gtrsim 10-20$), the error decays exponentially [see Supplemental Materials and ref. ^22^]. Then, we can estimate the firing probability ${\mathbb{P}}(v > \theta )=1-\Phi (\sqrt{3}\theta )$, where $\Phi (\,\cdot \,)$ is the normal cumulative distribution function. By using a binomial distribution, we get:

3 $$\begin{array}{l}{R}_{{\rm{slctv}}}=L\mathrm{(1}-\Phi (\sqrt{3}\theta ))\Phi {(\sqrt{3}\theta )}^{L-1},\\ {R}_{{\rm{inact}}}=\Phi {(\sqrt{3}\theta )}^{L},\\ {R}_{{\rm{lost}}}=\Phi {(\sqrt{3}\theta )}^{{m}_{s}}\mathrm{}.\end{array}$$

**Figure 2:**
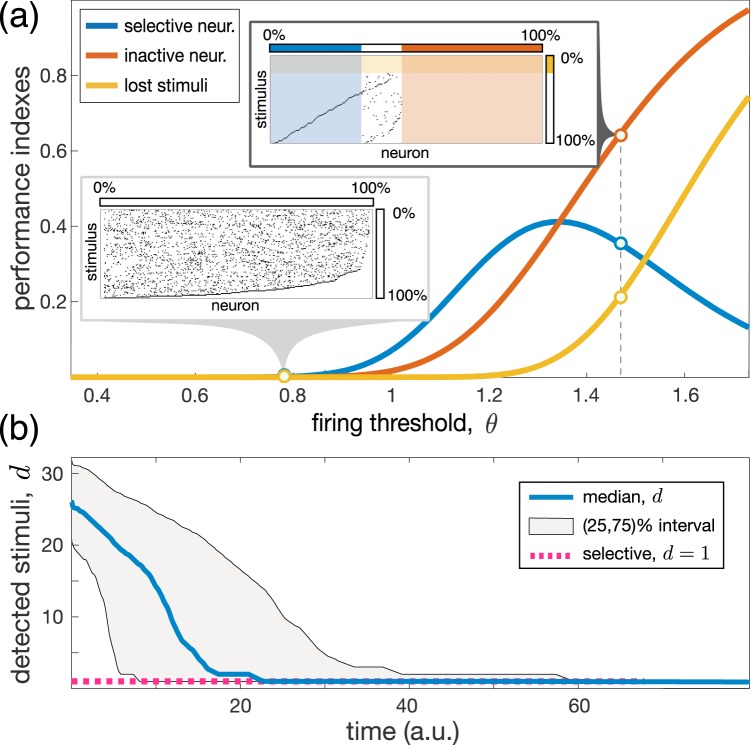
Poor initial brain performance and the emergence of selectivity through learning. **(a)** Performance indexes [Eq. (3), thick curves] of the selective stratum at $t=0$. Insets show raster plots of stimuli detected by neurons (${m}_{s}=300$ and $L=100$). **(b)** Median number of stimuli detected by neurons, $d$, *vs* time (${n}_{s}=30$, $L=400$, $\theta =0.5$, $\alpha =20$, and ${p}_{{\rm{sl}}}=0.95$).

Figure 2(a) illustrates the performance measures and two examples of raster plots of stimuli detected by neurons (in a raster plot a black dot at position $(i,\,j)$ means that neuron *i* detects stimulus *j*). The ratio of selective neurons ${R}_{{\rm{slctv}}}$ has a modest peak of height ${e}^{-1}$ at ${\theta }^{\ast }=\frac{1}{\sqrt{3}}{\Phi }^{-1}\left(\frac{L-1}{L}\right)\approx 1.35$. Therefore, at $t=0$ a randomly initialized selective stratum can have at most 37% of selective neurons, independently on the neuronal dimension *n*_*s*_. Thus, the first universal principle is:

• Different “brains” exhibit poor initial performance, regardless of the neuronal input dimension *n*_*s*_.

As we show now, learning can dramatically improve brain performance. We choose the firing threshold small enough (i.e., sufficiently lower than ${\theta }^{\ast }$), e.g., $\theta =1$. Then, with high probability, there are no inactive neurons nor lost stimuli (Fig. 2(a), ${R}_{{\rm{inact}}}\approx 0$, ${R}_{{\rm{lost}}}\approx 0$), i.e., Hebbian learning (2c) is activated for all neurons and all stimuli. Figure 2(b) illustrates the dynamics of the median number of stimuli detected by neurons. At $t=0$, all neurons in the aggregate respond in average to $d=25$ stimuli and hence are not selective, while at $t=80$ all of them are absolutely selective, $d=1$.

To extend this numerical observation, we first find the condition that a neuron, started firing to a stimulus ${{\bf{x}}}_{i}$, keeps firing in forward time with a probability no smaller than some constant $0 < {p}_{{\rm{sl}}} < 1$. This condition is fulfilled by choosing the order parameter (see Supplemental Materials):

4 $${\beta }_{{\rm{s}}{\rm{l}}}=\frac{\theta }{\delta },\,\delta =\sqrt{1-\frac{2{\Phi }^{-1}({p}_{{\rm{s}}{\rm{l}}})}{\sqrt{5{n}_{{\rm{s}}}}}}.$$

Note that the higher the neuronal dimension *n*_*s*_, the higher ${p}_{{\rm{sl}}}$ can be chosen. We also observe that if a neuron has the order parameter significantly lower ${\beta }_{{\rm{sl}}}$, then such a neuron “forgets” the stimulus ${{\bf{x}}}_{i}$ after a transient. In contrast, if $\beta$ is much higher ${\beta }_{{\rm{sl}}}$, then such a neuron cannot be selective. Thus, ${\beta }_{{\rm{sl}}}$ is the optimal order parameter for the selective stratum.

Under condition (4) the synaptic weights converge:

5 $${{\bf{w}}}_{\infty }:=\mathop{\mathrm{lim}}\limits_{t\to \infty }{\bf{w}}(t)={\beta }_{{\rm{sl}}}\frac{{{\bf{x}}}_{i}}{\Vert {{\bf{x}}}_{i}\Vert }\mathrm{}.$$

Thus, given that the relaxation time $\alpha$ is large enough, learning forces the neuron to “align” along its “preferable” stimulus: ${{\bf{w}}}_{\infty }\uparrow \,\uparrow {{\bf{x}}}_{i}$. At the same time, for high ${n}_{s}$, we have the property: $\langle {{\bf{x}}}_{i},{{\bf{x}}}_{k}\rangle \approx 0$, $i\ne k$ (for details see, e.g., refs. ^9,23^). Thus, after a transient, the neuronal membrane potential will be close to zero for all stimuli except ${{\bf{x}}}_{i}$ and hence the neuron will become selective.

To estimate the probability that a neuron will be selective after learning, i.e., $S:\,={\mathbb{P}}({d}_{j}=\mathrm{1)}$, we evaluate the probability that the neuron will be silent to another arbitrary stimulus ${{\bf{x}}}_{k}$ ($k\ne i$) (see Supplemental Materials):

6 $${p}_{a}={\int }_{0}^{\infty }\Phi (\delta \sqrt{{n}_{s}\xi })\kappa (\xi ;\mu ,\sigma )\,{\rm{d}}\xi ,$$

where $\kappa (\cdot \,;\,\mu ,\sigma )$ is the normal probability distribution function with the mean $\mu =1$ and the standard deviation $\sigma =\frac{2}{\sqrt{5{n}_{s}}}$. We note that with an increase of ${n}_{s}$, $\kappa$ concentrates around 1, and we can roughly evaluate ${p}_{a}\approx \Phi (\delta \sqrt{{n}_{s}})$, which rapidly tends to 1 for high *n*_*s*_. Finally, the neuronal selectivity,

7 $$S({n}_{s},L)={p}_{a}^{L-1},$$

depends on the number of stimuli *L* and the neuronal input dimension *n*_*s*_ only.

**Figure 3:**
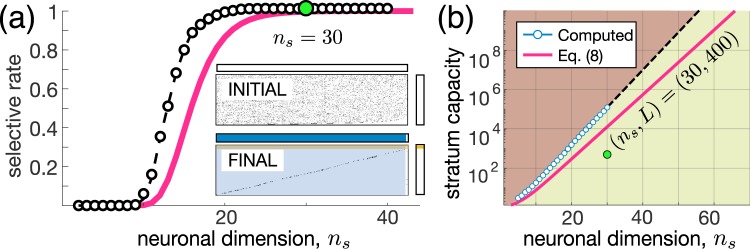
Emergence of extreme selectivity in high-dimensional brains. **(a)** Step-like increase of the ratio of selective neurons (experiment: black circles; estimate (7): red curve). Insets show raster plots of detected stimuli for ${n}_{s}=30$ (compare to Fig. 2(a)). **(b)** Exponential growth of the memory capacity (experiment: blue circles; estimate (8): red curve). Green area marks the working zone. Green dot corresponds to insets in (a).

Figure 3(a) shows the selectivity *S* as a function of the neuronal dimension *n*_*s*_. Learning yields a step-like dependence of *S* on *n*_*s*_. For small *n*_*s*_, there is no improvement of the selectivity by learning ($S\approx 0$), while for higher *n*_*s*_, it rapidly reaches 100%. Insets in Fig. 3(a) illustrate an example of raster plots of stimuli detected by neurons at the beginning and the end of learning. We observe that almost all neurons become selective to single information items (area shadowed by blue). Thus, the second universal principle is

- An increase in the neuronal input dimension *n*_*s*_ provokes an explosive emergence of selective behavior in “brains” composed of high-dimensional neurons at a critical dimension of 15–30. From Eq. (7) we can also estimate the maximal number of stimuli that a big enough stratum can work with:

8 $${L}_{{\rm{\max }}}\mathrm{=1}+\frac{\mathrm{ln}({p}_{L})}{\mathrm{ln}({p}_{a})},$$

where ${p}_{L}$ is the lower bound of the probability that the stratum detects all *L* stimuli. Figure 3(b) shows the theoretical and experimental estimates of the stratum capacity. Even for a rather moderate dimension ${n}_{s}=60$, the capacity goes beyond ${10}^{10}$ items (numerical estimate beyond ${n}_{s}=30$ was not calculated due to exponential growth of the computational load). In practical terms, it means that:

- A big enough “brain” consisting of high-dimensional neurons can selectively detect all stimuli existing in the world.

**Figure 4:**
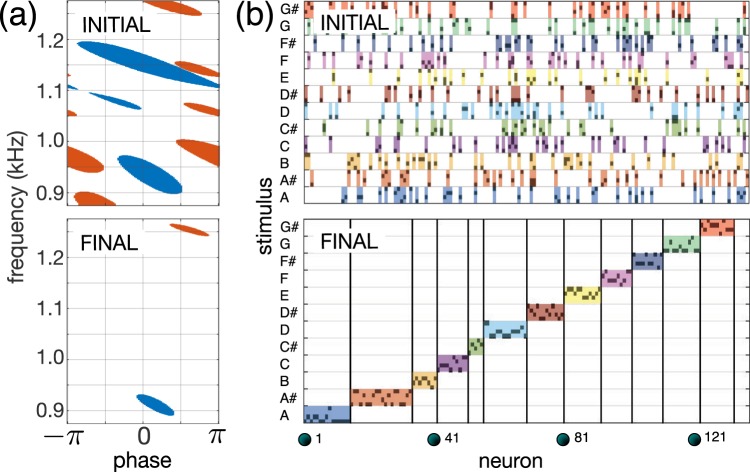
Learning musical stimuli by the selective stratum. **(a)** Receptive fields of two neurons in the “sound” space before and after learning. **(b)** Raster plot at $t=0$ (top) shows the random response of the stratum to 48 stimuli representing 12 musical notes from A to G#. After learning (bottom), neurons grouped into clusters selective to individual stimuli (sound waves).

To illustrate how the selective stratum can deal with “real-world” stimuli, we simulated learning of 48 sound waves corresponding to 12 musical notes from A to G# [see Eq. (1) and Fig. 1]. Figure 4(a) shows the receptive fields of two arbitrary chosen neurons in the selective stratum before and after learning. At the beginning, the neurons had wide random (even disjoint) receptive fields, as it was hypothesized at the beginning (see Fig. 1(b)). The learning reduced the receptive fields to tiny ellipses representing coherent stimuli (sound waves indistinguishable for neurons due to some finite tolerance). Thus, neurons in the selective stratum learn individual sound waves, and we observe the spontaneous formation of neuronal “clusters” (Fig. 4(b)). Individual neurons within a cluster detect sound waves with different phases corresponding to a single note while rejecting the other stimuli.

We also note that the size of clusters varies among different notes. It occurs due to the random initialization of the neurons, without *a priory* knowledge on the stimulus characteristics. A better allocation of the neurons across different stimuli can be achieved by introducing inhibitory inter-neuron couplings, which will prevent the emergence of large clusters responding to the same stimuli. Such an inhibitory mechanism is widely implemented in the hippocampus through multiple types of interneurons, which contribute up to 85% to the total power of local field potentials^24,25^.

### Emergence of concept cells

Let us now consider the second stratum composed of concept cells (Fig. 1(a)). The dynamics of concept cells is also described by Eq. (2) but now as an input we use the output from the selective stratum ${\bf{y}}\in {{\mathbb{R}}}_{+}^{{m}_{s}}$ within one time window:

9 $${{\bf{s}}}_{{\rm{int}}}(t)=\mathop{\sum }\limits_{i\mathrm{=1}}^{K}{{\bf{y}}}_{i}{\chi }_{i}(t),\,t\in [0,\,K\Delta ],$$

where ${\chi }_{i}(t)$ are overlapping rectangular time windows $[(i-\mathrm{1)}\Delta ,K\Delta )$. At the stratum output ${\bf{z}}$ (Fig. 1(a)), we then expect to obtain codification of concepts, which are associations of *K* individual stimuli.

The coding is now sparse. After learning, only a little portion of the neurons in the selective stratum responds to a single stimulus ${{\bf{x}}}_{i}$, i.e., $|{\rm{s}}{\rm{u}}{\rm{p}}{\rm{p}}({{\bf{y}}}_{i})|\ll {m}_{s}$. Thus,

- Sparse coding emerges naturally, without a predesigned structuring of the model.

The neuronal response ${{\bf{y}}}_{i}\ge 0$ and hence $\langle {{\bf{y}}}_{j},{{\bf{y}}}_{i}\rangle \ge 0$. Besides, after learning neurons in the first stratum are selective, i.e., ${d}_{j}=1$, $j=1,\ldots ,{m}_{s}$. Then, ${\rm{supp}}({{\bf{y}}}_{j})\cap {\rm{supp}}({{\bf{y}}}_{i})=\varnothing$ and hence $\langle {{\bf{y}}}_{j},{{\bf{y}}}_{i}\rangle =0$ ($j\ne i$), which facilitates learning in the concept stratum.

Repeating similar arguments for choosing the order parameter $\beta$ as provided above, after tedious calculations (see Supplemental Materials), we find:

10 $${\beta }_{{\rm{cn}}}=\frac{{\theta }_{{\rm{cn}}}\sqrt{L}\delta K\Gamma \left(K+\frac{1}{2}\right)}{{\theta }_{{\rm{sl}}}\mathrm{(1}-{p}_{{\rm{cn}}}\mathrm{)(1}-\delta )(K-\mathrm{1)!}\sqrt{{n}_{c}}},$$

where $\varGamma$ is the gamma function, and ${p}_{{\rm{c}}n}$ is the probability that after learning a neuron in the concept stratum will fire to all *K* stimuli, i.e., will become a concept cell. Equation (10) yields the following approximate condition on the neuronal dimension of CCs:

11 $${n}_{c}\propto {K}^{3}/{\beta }_{{\rm{cn}}}^{2}\mathrm{}.$$

An increase in the number of stimuli *K*, which can be associated with a concept, requires a cubic increase of the input dimension of the concept cells ${n}_{c}$, which can be balanced by the rise of the order parameter ${\beta }_{{\rm{cn}}}$. Thus, we have the following feature:

- The input dimension of the concept cells ${n}_{c}$ scales cubically with their association ability.

**Figure 5:**
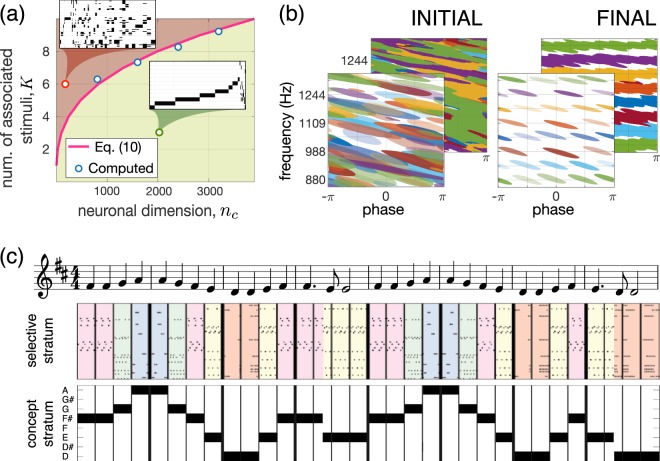
Emergence of concept cells and musical memory. **(a)** Working zone (shadowed by green) for association of stimuli in concepts. Insets show raster plots of the concept cells’ response: a random mixture in the red zone and correct association in the green zone. **(b)** Formation of “musical memory”. Receptive fields in the selective and concept strata are organized into wave- and note-specific structures, respectively [see also the hypothesis in Fig. 1(b)]. **(c)** Perception of a fragment of the 9-th Symphony by Beethoven “Ode to joy” ($K=7$, ${\theta }_{{\rm{sl}}}=1$, ${\theta }_{{\rm{cn}}}=0.1$, ${p}_{cn}=0.9$, $L=64$, ${n}_{s}=100$, ${m}_{s}=3200$, and ${m}_{c}=1600$; for visual clarity, in the middle subplot, the response of only 2% of the neurons in the selective stratum is shown).

Figure 5(a) shows how the association depth *K* scales with the neuronal dimension ${n}_{c}$. Overloaded associations with high *K* can result in the detection of “wrong” stimuli as being within a concept. Such an observation has been reported experimentally when a Jennifer Aniston neuron the next day also detected Lisa Kudrow from the TV series “Friends”^12^.

To illustrate the process of the formation of selective and concept cells for musical memory, we built a network consisting of ${m}_{s}=3200$ neurons in the selective and ${m}_{c}=1600$ neurons in the concept strata. These numbers ensure about 50 neurons for each learned stimulus in the selective layer spanning eight sound waves with different phase lags per each of the eight frequencies (notes D, D#, E, F, F#, G, G#, and A). Then, the used constraints define the depth of the concepts of musical notes $K=7$.

At the beginning, all selective and concept cells have messy receptive fields (Fig. 5(b), see also Fig. 4(a)). Learning organizes both strata as it was advanced in Fig. 1(b). We obtained about 85% of selective neurons in the first stratum. Since the association depth has been preselected ($K=7$ for eight phase lags), all neurons in the concept stratum ended up as concept cells.

We then tested the network in real conditions by simulating the process of perception of the 9th Symphony by Beethoven. Figure 5(c) shows the system response to a fragment of the symphony. As expected, the selective stratum detects individual sound waves, while the concept stratum puts them together and forms the note-specific output. Thus, concept cells respond to particular notes regardless of the phase of sound waves, and the “brain” now does follow the music.

## Discussion

In this work, we considered from the fundamental viewpoint the long-standing problem of the existence of concept cells in the human brain. Our findings have shown that the emergence of concept cells is conditioned by the synaptic (i.e., input) dimension of principal neurons in feedforward connected strata. This result has an important implication on the brain regions suspected to have CCs. For example, one of the anatomical requirements is the predominantly laminar organization of the neuronal strata with large modules of coherent activity produced by afferent pathways. Such a structural and functional organization facilitates the transmission of similar high-dimensional information to many postsynaptic cells as it happens in, e.g., the hippocampus^19^.

The evolution of living organisms and to some extent artificial neural networks towards more complex cognitive functionality requires an increase of the neuronal input dimension. A concept capable brain or an ANN should meet the following requirements: a) At least one selective and one concept strata; b) The adequate neuronal dimensions, e.g., ${n}_{s}\approx {10}^{2}$ and ${n}_{c}\approx {10}^{4}$ for the selective and concept layers, respectively; c) The order parameter *β* properly chosen for different strata.

We intentionally avoided the use of any *a priory* knowledge and constraints in the mathematical model. Thus, the conditions found are fundamental and have no specific relation to the fine features of the model used in our simulations. Encephalized animals and humans satisfy requirements (a) and (b). Thus, our results support the hypothesis of a strong correlation between the level of the neuronal connectivity in living organisms, and different cognitive behaviors such organisms can exhibit (cf. refs. ^3,26^). Condition (c) is related to the learning rate and hence to the magnitude of synaptic plasticity, which differs significantly among neurons^27^. It defines whether a neuron can be selective or associative.

We thus suggest a hierarchy of cognitive functionality. The first relay stations in the information processing, i.e., selective strata, gain extreme selectivity at intermediate dimensions (${n}_{s}\approx 30-100$). The second critical transition occurs at much higher dimensions ${n}_{c}\approx 500-1000$. Then, neurons located in the concept stratum become capable of associating multiple uncorrelated inputs of different sensory modalities into concepts. A straightforward extension of our model is an inclusion of more layers, which could encode the association of primary concepts into compound ones, as was observed experimentally^11^.

Recent experimental data^28^ suggest that neurons in the medial temporal lobe (including the hippocampus) codify high-level semantic abstractions at the population level. Then, the emergence of superordinate concepts (e.g., from a ‘dog’ to an ‘animal’) can be considered as a hierarchical generalization of knowledge codified by multiple concept cells. Our results indirectly support this hypothesis. We have shown that the required input dimension of the concept cells increases very fast (cubically) with the number of items associated with concepts. We then get a natural limitation on the associative ability of individual cells. Thus, high-level concepts must be fuzzy by their nature, and their construction may involve a hierarchical combinatorial composition of low-level categories, which can be experimentally observed at the population level.

Finally, the abstraction of “static” stimuli (objects, persons, landscapes, etc.) can be extended to the abstraction of actions and behaviors^29^. Our brain is capable of building and learning through observation of motor-motifs^30^ required for effective interaction with the environment. How neurons represent such spatiotemporal concepts is a challenge for further theoretical and experimental studies.

## Supplementary information

Supplementary information
