# Supplementary material for: Universal principles justify the existence of concept cells
Source: Sci Rep. 2020 May 12;10:7889. doi: 10.1038/s41598-020-64466-7 (PMC7217959; doi:10.1038/s41598-020-64466-7)
Supplement: Supplementary file 1 — Supplementary information [file 41598_2020_64466_MOESM1_ESM.pdf]

Supplemental material for:
Universal principles justify the existence of concept cells

Carlos Calvo Tapia, Ivan Tyukin, Valeri A. Makarov

1 Firing probability in selective stratum at $t = 0$

Before discussing the firing probability, let us mention some useful results.

1.1 Hoeffding's inequality

Let X_i, $i = 1, \ldots, n$ be random variables with compact support $\mathbb{P}(X_i \in [a, b]) = 1$, and $\bar{X} = \frac{1}{n}\sum X_i$. Then

$$\begin{aligned} \mathbb{P}(\bar{X} - E[\bar{X}] \geq t) \leq e^{-\frac{2nt^2}{(b-a)^2}} \\ \mathbb{P}(-\bar{X} + E[\bar{X}] \geq t) \leq e^{-\frac{2nt^2}{(b-a)^2}} \end{aligned} \tag{1}$$

for some $t > 0$ [1].

1.2 Central Limit Theorem

Let $\{X_i\}_{i=1}^n$ be n independent random variables with zero means and standard deviations $\{\sigma_i\}_{i=1}^n$. We introduce new random variable:

$$\hat{X} = \frac{\sum_{i=1}^n X_i}{\sqrt{\sum_{i=1}^n \sigma_i^2}} \tag{2}$$

with a certain cdf $F_n(\cdot)$. Then, we have [2]:

$$|F_n(\hat{X}) - \Phi(\hat{X})| \leq C \frac{\sum_{i=1}^n \rho_i}{\left(\sum_{i=1}^n \sigma_i^2\right)^{3/2}}, \tag{3}$$

where Φ is the cdf of the standard normal distribution and $\rho_i = E[|X_i|^3]$. Moreover, the constant, C, is bounded by [3, 4]:

$$0.4097 \simeq \frac{\sqrt{10}+3}{6\sqrt{2\pi}} \leq C \leq 0.56. \tag{4}$$

Property (3), being a version of the Central Limit Theorem, implies that empirical averages of independent random variables with zero means and finite second and third moments are asymptotically normally distributed as $n \to \infty$. If no further assumptions are imposed then the convergence rate is $O(1/\sqrt{n})$.

1.3 Decay of tails of the membrane potential

Employing (3), using a random $\boldsymbol{x}$ (see the main text), and noting that $\sigma^2 = E[x_i^2]E[w_i^2] = 1/9$ and $\rho = E[|x_i|^3]E[|w_i|^3] = 1/16$, the cumulative distribution of the membrane potential, v, satisfies

$$\left|F_n\left(\sqrt{3}v\right) - \Phi\left(\sqrt{3}v\right)\right| \leq \frac{27C}{16\sqrt{n}} \leq \frac{0.945}{\sqrt{n}} \tag{5}$$

where, as above, $F_n(\cdot)$ is the corrected distribution.

Using inequalities (1), we get the following estimate on the firing probability to a random stimulus $\boldsymbol{x}$:

$$\mathbb{P}(v > \theta) < e^{-\theta^2/6}. \tag{6}$$

Now by employing this concentration inequality together with (5), we find the bounds:

$$p_{\mathrm{dw}} \leq \mathbb{P}(v > \theta) \leq p_{\mathrm{up}}, \tag{7}$$

where

$$\begin{aligned} p_{\mathrm{up}} &= \min\left\{e^{-\theta^2/6}, 1 - \Phi(\sqrt{3}\theta) + \frac{0.945}{\sqrt{n}}\right\}, \\ p_{\mathrm{dw}} &= \max\left\{0, 1 - \Phi(\sqrt{3}\theta) - \frac{0.945}{\sqrt{n}}\right\}. \end{aligned} \tag{8}$$

For high n, these bounds converge to the probability value $1 - \Phi(\sqrt{3}\theta)$, provided in the main text. We also note the exponential convergence of $p_{\mathrm{up}}(\theta, n)$ to zero as a function of θ. This is a direct consequence of measure concentration effects.

2 Conditions of neuronal firing in forward time: Selection of $\beta_{\rm sl}$

We set the firing threshold small enough, e.g. $\theta = 1$. Then, with high probability, all neurons are active, i.e., $d_j \geq 1$, and there are no lost stimuli (Fig. 2(a) in the main text).

For convenience, we denote by $\boldsymbol{h} = \sqrt{\frac{3}{n}}\boldsymbol{x}_i$ the first stimulus activating the j-th neuron at $t^* \geq 0$, i.e., $y_j(t < t^*) = 0$, $y_j(t^*) > 0$. Let us now find the condition that the neuron keeps "firing" for $t > t^*$.

We decompose $\boldsymbol{w}_j$ into vectors parallel and orthogonal to $\boldsymbol{h}$ (by omitting the index j): $\boldsymbol{w} = \boldsymbol{w}_\parallel + \boldsymbol{w}_\perp$, where $\boldsymbol{w}_\parallel := q(t)\frac{\boldsymbol{h}}{\|\boldsymbol{h}\|}$ and $\langle \boldsymbol{w}_\parallel, \boldsymbol{w}_\perp \rangle = 0$. Then, Eq. (2c) from the main text yields:

$$\begin{aligned} \dot{\boldsymbol{w}}_\perp =& -\alpha\|\boldsymbol{h}\|yq\boldsymbol{w}_\perp, \\ \dot{q} =& \alpha\|\boldsymbol{h}\|y(\beta^2 - q^2). \end{aligned} \tag{9}$$

By construction, at $t = t^*$ the neuron fires, i.e., $v(t^*) = q(t^*)\|\boldsymbol{h}\| > \theta$. Note that $q(t \geq t^*) > 0$, otherwise $y = 0$ and there is no dynamics. Selecting $\beta > \theta/\|\boldsymbol{h}\|$ we ensure the firing condition $y(t \geq t^*) > 0$. Then, $\boldsymbol{w}_\perp(t) \to 0$ and $q \to \beta$, which implies:

$$\lim_{t\to\infty} \boldsymbol{w}(t) = \beta\frac{\boldsymbol{h}}{\|\boldsymbol{h}\|}, \tag{10}$$

provided in the main text.

Note that the value of β should not be too high, since it can diminish the neuronal selectivity (see below). Choosing $\beta = \theta/\|\boldsymbol{h}\| + \epsilon$, where $0 < \epsilon \ll 1$, ensures activity of the neuron but it requires knowledge of $\|\boldsymbol{h}\|$, inaccessible *a priori*. Then, by using $\|\boldsymbol{h}\|^2 \sim \mathcal{N}(1, \frac{2}{\sqrt{5n}})$ (directly follows from Section 1 for n high enough) and requiring $\mathbb{P}(\|\boldsymbol{h}\|^2 > \delta^2) = p_{\rm sl}$, where $\delta \in (0, 1)$ is a lower bound of $\|\boldsymbol{h}\|$, we can set:

$$\beta_{\rm sl} = \frac{\theta}{\delta}, \quad \delta = \sqrt{1 - \frac{2\Phi^{-1}(p_{\rm sl})}{\sqrt{5n}}}. \tag{11}$$

This guarantees faring of the neuron to the stimulus $\boldsymbol{h}$ in forward time with a probability no smaller than $p_{\rm sl}$. Note that the higher the neuronal dimension n, the higher $p_{\rm sl}$ can be chosen.

3 Selectivity after learning

We assume that a neuron has learnt an arbitrary stimulus, which we denote by $\boldsymbol{h} \in \left\{\sqrt{\frac{3}{n}}\boldsymbol{x}_i\right\}$. Then, after learning $\boldsymbol{w} = \beta\frac{\boldsymbol{h}}{\|\boldsymbol{h}\|}$. We now estimate the probability that the neuron is silent to another arbitrary stimulus $\boldsymbol{g} \in \{\sqrt{\frac{3}{n}}\boldsymbol{x}_i\}$ ($\boldsymbol{g} \neq \boldsymbol{h}$) given $\boldsymbol{h}$:

$$\mathbb{P}(y = 0|\boldsymbol{h}). \tag{12}$$

This can be done in several ways.

3.1 Probability by Hoeffding's inequality

From (12) we have:

$$\mathbb{P}(y = 0\,|\boldsymbol{h}) = \mathbb{P}\left(\langle \boldsymbol{h}, \boldsymbol{g}\rangle \leq \delta\|\boldsymbol{h}\|\,\middle|\boldsymbol{h}\right). \tag{13}$$

By employing the Hoeffding's inequality (1) we get

$$\mathbb{P}(\langle \boldsymbol{h}, \boldsymbol{g}\rangle \geq \tau) \leq e^{-n\tau^2/18}, \tag{14}$$

and hence

$$\mathbb{P}(y = 0\,|\boldsymbol{h}) > 1 - e^{-n\|\boldsymbol{h}\|^2\delta^2/18}. \tag{15}$$

Now, by recalling $\|\boldsymbol{h}\|^2 \sim \mathcal{N}(1, \frac{2}{\sqrt{5n}})$ for high enough n, we obtain:

$$\mathcal{P}_H = \mathbb{P}(y = 0) > 1 - e^{-\gamma(n)}. \tag{16}$$

where $\gamma(n) = \frac{\delta^2 n}{18}(1 - \frac{\delta^2}{45})$ is an increasing function of n.

3.2 Probability by normal distribution

By employing normal distribution, from (12) we get:

$$\mathbb{P}(y = 0|\boldsymbol{h}) = \Phi(\delta\|\boldsymbol{h}\|\sqrt{n}). \tag{17}$$

Then, we extend it to arbitrary $\boldsymbol{h}$ as above:

$$\mathcal{P}_N = \int_0^\infty \Phi(\delta\sqrt{ns})\kappa(s; \mu, \sigma)\,\mathrm{d}s, \tag{18}$$

where $\kappa(\cdot; \mu, \sigma)$ is the normal pdf with the mean $\mu = 1$ and the standard deviation $\sigma = \frac{2}{\sqrt{5n}}$. Equation (18) corresponds to Eq. (6) in the main text.

3.3 Comparison of two approaches

The neuronal selectivity is given by [Eq. (7) in the main text] $S(n, L) = \mathcal{P}^{L-1}$, where $\mathcal{P}$ can be taken either from (16) or from (18). Figure 1 shows the neuronal selectivity estimated by two methods. The lower bound estimated from inequalities (16) (blue curve) is too conservative, while Eq. (18) matches well the numerical results (see Fig. 3(a) in the main text).

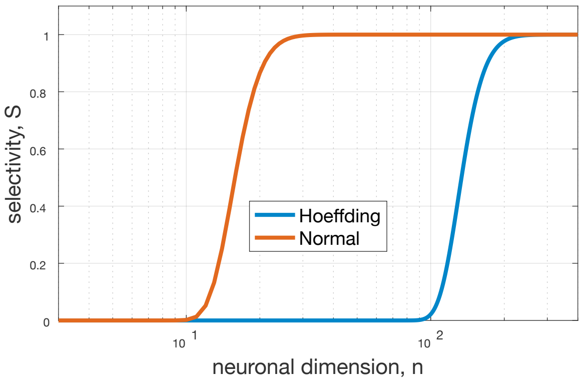

Figure 1: Two estimates of S (see also Fig. 3(a) in the main text, all parameter values are the same).

4 Order parameter β_{cn} for concept stratum

A neuron in the concept stratum receives as an input the stimulus $\boldsymbol{h}_k = \sum_{i=1}^{k} \boldsymbol{y}_i$, where k defines the time window (see Eq. 9 in the main text).

4.1 Learning condition

At $t = 0$, we assume that the neuron detects the first stimulus $\boldsymbol{h}_1 = \boldsymbol{y}_1$, i.e., $\langle \boldsymbol{w}(0), \boldsymbol{h}_1 \rangle > \theta_{\text{cn}}$, which is equivalent to $q(0) > \theta/\|\boldsymbol{h}_1\|$ in Eq. (9). Thus, to keep firing we require

$$\beta_{\text{cn}} > \frac{\theta_{\text{cn}}}{\|\boldsymbol{h}_1\|}. \tag{19}$$

By using Eq. (9) we get that, at the end of the first interval Δ, $\boldsymbol{w} \to \beta_{\mathrm{cn}}\boldsymbol{h}_1/\|\boldsymbol{h}_1\|$. In general, the initial condition for the k-th interval is

$$\boldsymbol{w}_{k0} = \lim_{t\to(k-1)\Delta} \boldsymbol{w}(t) \approx \beta_{\mathrm{cn}}\frac{\boldsymbol{h}_{k-1}}{\|\boldsymbol{h}_{k-1}\|}. \tag{20}$$

This is equivalent to

$$q_{k0} = \beta_{\mathrm{cn}}\frac{\langle \boldsymbol{h}_{k-1}, \boldsymbol{h}_k\rangle}{\|\boldsymbol{h}_{k-1}\|\|\boldsymbol{h}_k\|}. \tag{21}$$

To meet the firing condition at $t = (k-1)\Delta$, we require $q_{k0} > \theta_{\mathrm{cn}}/\|\boldsymbol{h}_1\|$ which yields

$$\beta_{\mathrm{cn}} > \frac{\theta_{\mathrm{cn}}}{\|\boldsymbol{h}_1\|}\frac{\|\boldsymbol{h}_k\|}{\|\boldsymbol{h}_{k-1}\|} > \frac{\theta_{\mathrm{cn}}}{\|\boldsymbol{h}_1\|}, \tag{22}$$

where we used $\langle \boldsymbol{y}_i, \boldsymbol{y}_j\rangle = 0$ for $j \neq i$ (see the main text). Thus, given that α is big enough, the neuron will fire during the whole process of learning.

Once the learning is finished, $\boldsymbol{w} = \beta_{\mathrm{cn}}\frac{\boldsymbol{h}_K}{\|\boldsymbol{h}_K\|}$. Then, the neuron is a concept cell if

$$\beta_{\mathrm{cn}} > \frac{\theta_{\mathrm{cn}}\|\boldsymbol{h}_K\|}{\|\boldsymbol{y}_i\|^2}, \quad i = 1, 2, \ldots, K, \tag{23}$$

which is equivalent to

$$\beta^2 > \theta_{\mathrm{cn}}^2\frac{\sum_{i=1}^K \|\boldsymbol{y}_i\|^2}{\min_{i\in\{1,\ldots,K\}}\{\|\boldsymbol{y}_i\|^4\}}. \tag{24}$$

4.2 Estimate of β_{cn}

For convenience, let's denote:

$$S = \sum_{i=1}^K \|\boldsymbol{y}_i\|^2, \quad M = \min_i\{\|\boldsymbol{y}_i\|^2\}. \tag{25}$$

We then set $\beta_{\mathrm{cn}} = \theta_{\mathrm{cn}}\Psi$, where Ψ satisfies [Eq. (24)]:

$$\mathbb{P}(M^2\Psi^2 > S) = p_{\mathrm{cn}}, \tag{26}$$

where p_{cn} is the lower probability bound. This equation ensures that the concept stratum learns at least K inputs with the probability not smaller than p_{cn}.

For further calculations, we assume that $z := \|\boldsymbol{y}\|^2$ is exponentially distributed:

$$f_z(z) = \begin{cases} \lambda e^{-\lambda z}, & z > 0 \\ 0 & \text{otherwise} \end{cases} \tag{27}$$

for some constant $\lambda > 0$. Then, S follows the Erlang distribution:

$$f_S(s) = \frac{\lambda^K s^{K-1} e^{-\lambda s}}{(K-1)!}, \quad s > 0. \tag{28}$$

To find the distribution of M we write:

$$F_M(m) = \mathbb{P}(\min\{z_i\} \leq m) = 1 - (1 - F_z(m))^K, \tag{29}$$

where F_z is the cdf of z. Thus,

$$F_M(m) = 1 - e^{-K\lambda m}, \quad m > 0. \tag{30}$$

We now can assume that S and M are independent and hence $f(m,s) = f_M(m) f_S(s)$. Then, Eq. (26) yields

$$p_{\mathrm{cn}} = \int_0^\infty f_S(s)(1 - F_M(\sqrt{s}/\Psi))\,\mathrm{d}s. \tag{31}$$

By using (28), (30), (31), and operating, we get

$$p_{\mathrm{cn}} = \int_0^\infty \frac{u^{K-1} e^{-u-a\sqrt{u}}}{(K-1)!}\,\mathrm{d}u, \quad a = \frac{K\sqrt{\lambda}}{\Psi}. \tag{32}$$

We now note that a is a small parameter. Thus, we can approximate $e^{-u-a\sqrt{u}} \approx e^{-u}(1 - \sqrt{u}a)$ and evaluate the integral (32):

$$p_{\mathrm{cn}} = 1 - a\frac{\Gamma(K + \frac{1}{2})}{(K-1)!}. \tag{33}$$

This equation provides the estimate:

$$\beta_{\mathrm{cn}} = \theta_{\mathrm{cn}} \sqrt{\lambda} \frac{K\Gamma(K + \frac{1}{2})}{(1 - p_{\mathrm{cn}})(K-1)!}. \tag{34}$$

We now note that $\lambda = 1/E[z]$ and assume that all neurons in the selective stratum have learnt stimuli, i.e.,

$$z = \|(\beta_{\mathrm{sl}}\|\boldsymbol{h}\| - \theta_{\mathrm{sl}})\boldsymbol{b}\|^2, \tag{35}$$

where $\boldsymbol{b}$ is a binary vector representing neurons activated by the stimulus $\boldsymbol{h}$. Thus,

$$E[z] = \beta_{\mathrm{sl}}^2 E[(\|\boldsymbol{h}\| - \delta)^2] E[\|\boldsymbol{b}\|^2]. \tag{36}$$

Then, we note that $\|\boldsymbol{b}\|^2 \sim \mathcal{B}(m,p)$ and hence $E[\|\boldsymbol{b}\|^2] = mp$. In the case that all L stimuli have been learnt, we have $p = L^{-1}$. Now, we have $E[(\|\boldsymbol{h}\| - \delta)^2] = 1 - 2\delta E[\|\boldsymbol{h}\|] + \delta^2$. In the first order approximation $E[\|\boldsymbol{h}\|] \approx 1$. Thus, we have

$$\lambda \approx \frac{L}{\beta_{\text{sl}}^2 (1-\delta)^2 m}. \tag{37}$$

Substituting approximation (37) into Eq. (34) we obtain Eq. (10) provided in the main text.

References

[1] W. Hoeffding, Probability inequalities for sums of bounded random variables. *J. Am. Stat. Assoc.* **58**, 13-30 (1963).

[2] C.G. Esseen, On the Liapunoff limit of error in the theory of probability. *Arkiv för Mat., Astr. & Fys.* **A28**, 1-19 (1942).

[3] C.G. Esseen, A moment inequality with an application to the central limit theorem. *Skand. Aktuarietidskr.* **39**, 160-170 (1956).

[4] I.G. Shevtsova, An improvement of convergence rate estimates in the Lyapounov theorem. *Dokl. Math.* **82**, 862-864 (2010)
